# Hypermobility and chronic pain in adolescents: diverging functional and neural profiles without sensory differences

**DOI:** 10.1097/j.pain.0000000000003999

**Published:** 2026-05-15

**Authors:** Katrina Huft, Emma F. Gaydos, Jewel N. White, Saül Pascual-Diaz, Elke Schubert-Hjalmarsson, Natasha Harrison, Lita Moua, Marie-Eve Hoeppli, Emma Biggs, Jennifer N. Stinson, Nima Aghaeepour, Martin S. Angst, Brice Gaudilliere, Robert C. Coghill, Massieh Moayedi, Marina López-Solá, Christopher D. King, Laura E. Simons

**Affiliations:** a Duke University School of Medicine, Durham, NC, United States; b Department of Anesthesiology, Perioperative and Pain Medicine, Stanford University School of Medicine, Palo Alto, CA, United States; c School of Medicine, University of Barcelona. Institute of Neuroscience, University of Barcelona. IDIBAPS, Hospital Clinic, Barcelona, Spain; d Division of Physiotherapy, Department of Health and Rehabilitation, Institute of Neuroscience and Physiology, Sahlgrenska Academy, University of Gothenburg, Gothenburg, Sweden; e Stanford University, Palo Alto, CA, United States; f Cincinnati Children's Hospital Medical Center, Department of Pediatrics, Division of Behavioral Medicine and Clinical Psychology, Cincinnati, OH, United States; g Research Institute, The Hospital for Sick Children, Toronto, ON, Canada; h Centre for Multimodal Sensorimotor and Pain Research, Faculty of Dentistry, University of Toronto, Toronto, ON Canada

**Keywords:** Chronic pain, Pediatric pain, Ehlers-Danlos syndrome, Hypermobility, Magnetic resonance imaging, Quantitative sensory testing

## Abstract

Chronic pain (CP) affects approximately 20% of children, significantly impacting their physical, emotional, and social well-being. Hypermobility disorders (HDs) are prevalent in pediatric populations, including those with CP, and often contribute to impairments in psychological and physical functioning. This study examined differences in sensory, functional, and neural circuitry among CP in youth with and without comorbid HD (CP + HD). Contrary to our a priori hypothesis and previous literature suggesting that youth with CP + HD would exhibit greater suprathreshold pain sensitivity and lower pain thresholds, our findings revealed no significant differences in quantitative sensory testing measures between the 2 groups. This suggests that heightened pain sensitivity observed in CP + HD cannot be solely attributable to their hypermobility. However, the CP + HD cohort reported greater pain spread, increased fatigue, and poorer sleep quality, indicating that while they may not be more vulnerable to pain sensitization, they experience greater overall impairment in health and functioning. Furthermore, site-stratified analyses of multisensory task-evoked fMRI data revealed group differences in cerebellar activation and baseline cerebellar-supplementary motor cortex connectivity in youth with CP + HD, indicating altered neural strategies for sensory-motor integration. Notably, these significant fMRI findings were observed at 1 of the 2 study sites. Our results underscore the need for a nuanced understanding of the interplay between hypermobility, CP, and functional impairment to inform targeted interventions that address the multifaceted challenges faced by these patients.

## Abstract 2

Supplemental Digital Content is Available in the Text.

Youth with chronic pain and hypermobility show greater functional impairment and altered neural activity, but not increased sensitization, compared with those with chronic pain alone.

## 1. Introduction

Chronic pain (CP), defined as pain lasting ≥3 months,^55,87^ is common in the pediatric population, affecting approximately 1 in 5 children^14^ and significantly impacting well-being.^88^ Hypermobility disorders (HDs), including hypermobile Ehlers-Danlos Syndrome and Hypermobility Spectrum Disorder, are heritable connective tissue disorders where chronic pain contributes to functional impairment^71,91^ and can have profound physical and psychological impacts.^33^ In pediatric patients, the burden of HD may be particularly severe,^2,72^ exacerbated by pain, fatigue, gastrointestinal disorders, and dysautonomia.^63,86^ CP is associated with sequelae, including impaired physical function, poor mental health, and sleep disturbances, substantially impacting daily activities and quality of life.^7,26,40,89^ Patients with HD may experience this to a greater degree, with higher pain interference, fatigue, and sleep problems compared with healthy controls and individuals with other CP conditions.^17,57,59,63,72,80,92^ This compounded burden suggests that living with both CP and HD further complicates overall health.

Beyond functional challenges, patients with HD exhibit distinct pain processing characteristics, such as lower pressure-pain thresholds,^68,73^ higher temporal summation of pain,^46^ hyperalgesia to cold and hot temperatures, and lower exercise-induced hypoalgesia,^23^ indicating increased central sensitization.^46^ Similar quantitative sensory testing (QST) profiles are documented across CP populations.^76,85,95^ Consequently, prior studies of QST in HD may not adequately distinguish sensory alterations unique to HD from those common to CP broadly. This uncertainty is especially pronounced in pediatric populations, where research remains limited.

Neuroimaging data could enhance our understanding of CP and comorbid HD. Prior studies have elucidated the structural and functional brain alterations in CP, identifying regions including the thalamus, insula, and prefrontal cortex as integral to pain perception and sensory integration.^61,83,93^ Research has highlighted sensory processing differences in CP, particularly in accurately decoding and responding to sensory stimuli.^67^ In HD, existing studies have primarily concentrated on psychiatric symptoms,^28,41^ autonomic function,^27^ or physical trauma.^36^ Research examining neuroimaging correlates among patients with HD and comorbid CP remains limited, which poses a gap in the literature.

Given the limited understanding of the consequences of living with CP and HD in pediatric populations, this study aims to isolate HD's influence on the sensory experience. We aim to achieve this by comparing QST findings between 2 groups: (1) young people with CP *with* comorbid HD, and (2) those *without* comorbid HD. We will also evaluate brain mechanisms associated with sensory abnormalities across groups using neuroimaging paradigms that assess multisensory processing. Furthermore, we aim to understand how these differences are associated with pain-related physical and mental health (eg, pain spread, sleep quality, fatigue). We hypothesize that those with CP and comorbid HD will experience greater sensitization and functional impairment compared with those with CP alone. We further anticipate that these differences will manifest in neural activity of key sensory integration regions. Characterizing these clinical, behavioral, and neurobiological correlates can guide interventions that have the potential to advance precision care, whereby rehabilitation and pain management strategies are tailored to maximize functional recovery and quality of life.

## 2. Methods

### 2.1. Participant recruitment and categorization

Participants were recruited from Stanford University, Cincinnati Children's Hospital, and Toronto SickKids as part of the SPRINT study (NCT04285112), a larger biomarker study evaluating the signatures of pain recovery in adolescents with chronic pain.^74^ Adolescents with a diagnosis of chronic musculoskeletal pain were enrolled. For inclusion, the adolescent (age 10-18) must have had a diagnosis of chronic primary musculoskeletal pain (other than orofacial) derived from ICD-10. Patient exclusion criteria were (1) significant cognitive impairment (eg, severe brain injury), (2) claustrophobia, (3) significant medical or psychiatric problem that would interfere with this study (eg, seizures, psychosis), (4) pregnancy, (5) MRI contraindication, and (6) current or past opioid use >1 month.

**Figure 1.**
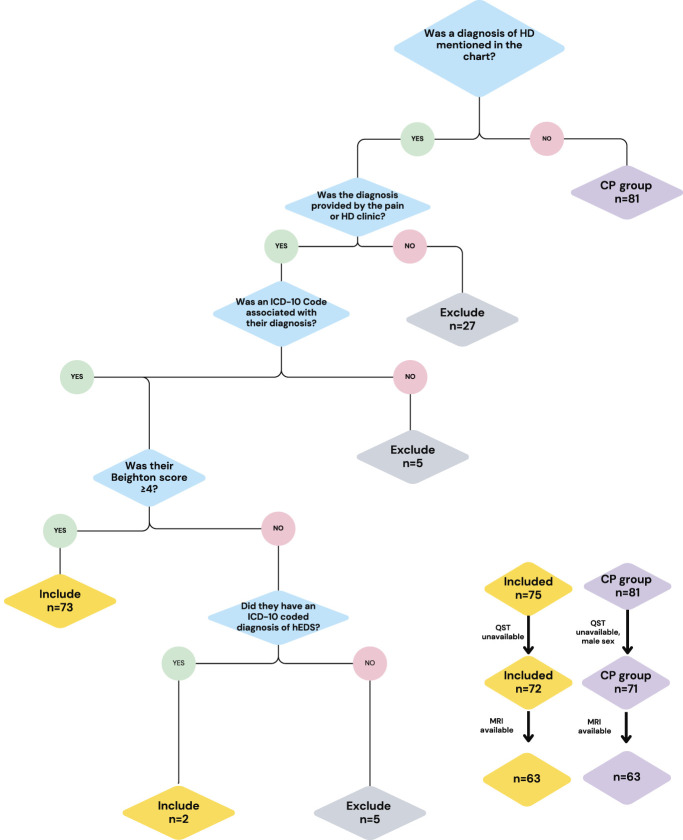
Flowchart illustrates the decision tree used for patient selection and analysis. The flowchart delineates the inclusion and exclusion criteria for participants diagnosed with HD and those in the control group (CP without HD). The final count of participants included in the HD group vs the control group is indicated at the bottom right of the flowchart. “QST unavailable” and “male sex” refer to the exclusion of participants who had insufficient QST data and those of male sex assigned at birth in the control (CP) group to match the female majority of the HD group. QST, quantitative sensory testing.

For the purpose of this study, only adolescents from the Stanford and Cincinnati sites were included, as the Toronto SickKids pain clinic does not generally evaluate hypermobility disorders. In total, 193 participants were enrolled in the SPRINT study across the Stanford and Cincinnati sites. Of these 193 participants, a thorough chart review through provider notes was conducted to sort participants into 1 of 3 groups: those with chronic MSK pain and a comorbid hypermobility disorder (HD group), those with chronic MSK pain without comorbid HD (CP group), or those with unclear hypermobile status (exclusion group). We used the Beighton score^49,77^ as a metric for joint hypermobility in our decision tree, which is scored on a scale of 0 to 9, with higher scores indicating more joint hypermobility. The details for the inclusion and exclusion criteria for these groupings are below, as represented in Figure 1.

Of note, the cutoff for inclusion based on the Beighton score of 4 was chosen as this is a commonly used cutoff for adults with HD. Children do not have a universally agreed-upon cutoff, though a score of 6/9 has been proposed for children and biologically immature adolescents.^86^ Ninety-three percent of our sample with HD was postpubertal (n = 67) and, therefore, had likely reached biological maturity. Similarly, 98.6% of our CP sample without HD was also postpubertal (n = 70). Given this vast majority, we opted for a lower cutoff, which is proposed to be appropriate for use in biologically mature adolescents.^86^ Notably, the cutoff values for the Beighton score have varied over time,^34^ and therefore, the presence of an ICD-10 coded HD diagnosis was considered more important for our purposes. Having said this, for thoroughness, a sensitivity analysis of the QST measures was performed to assess the robustness of our findings, specifically in participants with a Beighton score of ≥ 6. This analysis aimed to determine whether excluding individuals with lower hypermobility scores affected the overall results.

### 2.2. Procedures

This protocol was approved by the Cincinnati Children's Institutional Review Board (sIRB number 2019-1006), which served as the Single IRB for this study. Survey data were collected using electronic data capture by REDCap.^37^ REDCap surveys were deployed using a secure link or QR code to the participants' email addresses and phone numbers. Surveys were distributed 24 hours before their baseline visit. During their baseline visit, participants completed 3 tasks: blood draw, MRI, and QST. Full study visit procedures can be found in the original protocol paper.^74^ Diagnosis data were extracted by clinical research staff from patient medical charts on Epic.

### 2.3. Demographics, pain-related characteristics, and self-reported outcomes

Demographic data collected included age, sex, race, and ethnicity. Pubertal development was measured as an average across responses on the Pubertal Development Scale.^65^ Pain-related characteristics reported included pain duration, pain intensity, pain unpleasantness, and pain spread. Pain intensity and unpleasantness were measured on a visual analog scale (VAS); VAS ratings of pain intensity or unpleasantness “in the past week” involved moving a slider on a continuum from none to most, without visible numerical values. Pain spread was measured as the self-selected number of pain zones (out of 7) on a clickable body map.

Self-report measures were also collected:

#### 2.3.1. Brief Pain Inventory

The Brief Pain Inventory^19^ short form was used to measure pain's impact on functioning. We specifically analyzed the pain interference subscale of the Brief Pain Inventory. This 7-item measure evaluates how pain has interfered with activities (eg, walking, mood, sleep) in the past 7 days. Higher scores indicate more interference.

#### 2.3.2. Functional Disability Inventory

Functional impairment was measured using the Functional Disability Inventory,^15^ which consists of 15 items rated on a 5-point Likert scale from 0 (no trouble) to 4 (impossible to do). Higher scores represent more functional impairment.

#### 2.3.3. Fear of pain

Fear of pain is measured using the Fear of Pain Questionnaire short form,^39,75^ which is a 10-item questionnaire that assesses pain-related fear on a scale from 0 (strongly disagree) to 4 (strongly agree). Higher scores indicate more fear of pain.

#### 2.3.4. Patient-Reported Outcomes Measurement Information System measures

The Patient-Reported Outcomes Measurement Information System (PROMIS) was established by the National Institutes of Health to offer clinicians and researchers validated tools for assessing patient-reported outcomes across various areas of physical, mental, and social health, thereby enhancing consistency among studies. The survey items utilize a Likert scale ranging from 1 (never or unable to do) to 5 (almost always or with no difficulty). Scores are subsequently derived from a T-score distribution. The pediatric measures have been validated for children aged 8 to 17 years.^24,42,51^

##### 2.3.4.1. Patient-Reported Outcomes Measurement Information System pediatric fatigue

The PROMIS pediatric fatigue 10-item questionnaire^80^ evaluates the impact of fatigue on daily cognitive, emotional, and social functioning (eg, “I was too tired to pay attention,” “I got tired easily”) over the past 7 days. Higher scores demonstrate worse fatigue.

##### 2.3.4.2. Patient-Reported Outcomes Measurement Information System depression

Depression was measured using the PROMIS Depression short form, which is an 8-question survey scored on a scale from 0 (never) to 4 (almost always) containing prompts such as “I could not stop feeling sad” or “I felt alone” in the past 7 days. Higher scores represent worse depression.

##### 2.3.4.3. Patient-Reported Outcomes Measurement Information System anxiety

Anxiety was measured on a scale of 0 (never) to 4 (almost always) through statements including “I felt worried” or “I got scared easily” in the past 7 days. Higher scores indicate more anxiety.

#### 2.3.5. Pediatric Quality of Life

Quality of life was measured using the Pediatric Quality of Life short form^78^ which is scored on a scale of 0 (never a problem) to 4 (almost always a problem) across domains of physical, emotional, social, and school functioning. Higher scores indicate a better quality of life.

#### 2.3.6. Pain catastrophizing

Pain catastrophizing was measured using the Child Pain Catastrophizing Scale,^21^ which is a 13-item questionnaire that assesses increased negative cognitive and emotional reactions to pain on a scale from 0 (not at all true) to 4 (very true). Higher scores indicate more pain catastrophizing.

#### 2.3.7. Sleep quality

Sleep quality was measured using the Adolescent Sleep Wake Scale short form,^11,82^ which is a 10-item measure on a scale from 1 (always) to 6 (never), with higher scores indicating better sleep quality.

### 2.4. Quantitative sensory testing

A QST-trained examiner conducted all sensory assessments. The patient was first seated comfortably in a chair. A standardized set of instructions was provided, and a practice trial was demonstrated on an alternative, nonpainful area of the body (eg, nondominant hand). Patients were asked to report their painful body regions using a body map and indicate their single most affected pain site (MAS). If the indicated MAS was difficult to test or the participant did not consent to testing on this region, the second-most affected pain site was used.

The standardized testing sequence for QST included the assessment of the following parameters:

#### 2.4.1. Mechanical detection threshold

Mechanical detection threshold (MDT) was assessed with an ascending and descending series of von Frey monofilaments, beginning with the lightest stimulus (0.63, 1.37, 3.14, 16.67, 50.00, 81.40, and 235.36 mN). Von Frey monofilaments were applied perpendicular to the skin site for approximately 2 seconds with a force sufficient to cause a slight bend or S-shape in the filament. Participants were instructed to close their eyes and say “yes” at each felt stimulus. An up-down method was used such that “yes” responses were followed by a systematic decrease in stimulus intensity until no response was achieved, and no-response trials prompted a systematic increase in stimulus intensity until a “yes” response was achieved. Appearance and disappearance thresholds were calculated as the mean of responses. This procedure was conducted on both the MAS and the dorsal side of the nondominant hand. The starting site was randomized.

#### 2.4.2. Mechanical pain threshold

Mechanical pain threshold was assessed with an ascending sequence of weighted pinprick stimulators with standardized intensities (8, 16, 32, 64, 128, 256, and 512 mN). Pinprick stimulators were applied with a flowing movement and a 1-second contact duration. Participants were instructed to close their eyes and to say “yes” to indicate pain and “no” to indicate no pain. Moreover, participants were directed to consider pricking, stinging, or burning sensations as painful. Similar to the MDT, an ascending and descending process was used, and the mean was calculated to identify pain thresholds. Mechanical pain threshold was assessed on the dorsal side of the nondominant hand and the MAS. The starting site was randomized.

#### 2.4.3. Mechanical pain sensitivity

Mechanical pain sensitivity (MPS) was similarly assessed, and the patient was asked to report their pain intensity and unpleasantness on a VAS in response to a semi-randomized sequence of pinprick weights (8, 16, 32, 64, 128, 256, and 512 mN) in a balanced order. Participants were instructed to close their eyes during pinprick stimulations. In total, each of the 7 stimulus intensities was applied 4 times. Sensitivity to each intensity was calculated as the mean of pain intensity and unpleasantness scores across all 4 stimulations with that intensity. MPS was conducted on the nondominant ventral forearm only.

#### 2.4.4. Pressure pain threshold

Pressure pain threshold (PPT) was assessed with a hand-held algometer (Algomed, Medoc) across 3 repetitions of stimuli applied perpendicularly to bilateral trapezius muscles, thenar muscles, and the knee medial joint lines. A feedback system was incorporated to ensure a standardized pressure increase of 60 kPa/s. Pressure did not exceed 900 kPa. Participants were instructed to press a button and say “pain” when the stimulus first became painful. The button prompted the feedback system to record the current pressure. The mean of the 3 repetitions was taken at each location to assess pain intensity by body region. The starting side (right, left) of the body was randomized.

#### 2.4.5. Temporal summation of pain

Temporal summation of pain was assessed using a 2-stage stimulation procedure. Initially, each participant received a single stimulus from a 256-mN weighted pinprick stimulator, after which they were asked to rate their pain intensity and unpleasantness on the VAS scale. Following this initial rating, the same site was subjected to a series of 10 consecutive stimuli with 1 stimulus per second. Immediately after this repeated stimulation, participants were again prompted to rate their pain intensity and unpleasantness using the same scale. This process was repeated 3 times on the nondominant ventral forearm and 3 times on the MAS. The starting site was randomized. Each repetition was spaced apart by 2 minutes to prevent carry-over effects. Temporal summation was calculated by subtracting the average pain rating following 10 repeated stimuli from the average pain rating following a single stimulus.

#### 2.4.6. Conditioned pain modulation

Conditioned pain modulation (CPM) was evaluated by assessing changes in baseline PPT (before hand immersion), during (at 40 seconds following initial immersion), and after (at 70 seconds following initial immersion) hand immersion in a 10°C circulating water bath (Techne TE-10D Thermoregulator, B-8 Bath, and RU-200 Dip Cooler; Techne, Burlington, NJ). Moreover, participants reported pain intensity and unpleasantness from the cold-water bath on a VAS scale at 30 seconds and 60 seconds of submersion. Participants were prompted to remove their hand from the cold water at 60 seconds of submersion. The nondominant trapezius was used as the test stimulus site, and the contralateral hand's cold pressor test was the conditioning stimulus. This ensured adequate distance between the test (PPT) and conditioning (water bath) stimuli. The outcome measures for the CPM were obtained by subtracting the baseline PPT (pre-immersion) from the PPT measured during immersion or post-immersion. A positive change score indicated an increased pain threshold, signaling activated pain inhibition, while a negative or no change suggested impaired inhibitory function.

#### 2.4.7. Cold pain tolerance

Cold pain tolerance was assessed with an 8°C circulating water bath (Techne TE-10D Thermoregulator, B-8 Bath, and RU-200 Dip Cooler; Techne, Burlington, NJ). Participants immersed their hand up to the wrist, with the palm facing down and fingers spread apart, for a maximum of 180 seconds. If the pain became too intense, they could remove their hand earlier. Participants were instructed to notify the experimenter when the cold stimuli first became painful and to keep their hand submerged as long as possible. Pain start time and total submersion time were recorded.

### 2.6. Magnetic resonance imaging

A total of 126 participants underwent MRI scanning for this study, comprising 63 individuals with CP with comorbid HD (CP + HD group) and 63 with CP without comorbid HD (CP group). Eighty participants were scanned at Stanford University (32 in the CP + HD group and 48 in the CP group) using a GE Premier scanner. In total, 46 participants were scanned at Cincinnati Children's Hospital (31 in the CP + HD group and 15 in the CP group) using a Philips Ingenia Elition.

All 126 participants who underwent MRI completed the same multisensory fMRI task.^47,48^ The repetition time was 1500 milliseconds at both sites, with an echo time of 30 milliseconds at Stanford University and 35 milliseconds at Cincinnati Children's Hospital. The flip angle was 70°, and the field of view was 220 mm at both sites. Matrix sizes were standardized to 88 × 88 (Stanford) and 88 × 87 (Cincinnati), with 2.5 mm^3^ voxels and 57 slices per volume. In-plane acceleration techniques varied based on scanner capabilities, with parameters of Acceleration phase = 2 on the GE Premier and SENSE = 1 on the Philips Ingenia Elition. A multiband factor of 3 was consistently applied to all scans, resulting in 174 volumes per functional time series.

The multisensory task consisted of 4 trials, each containing alternating periods of multisensory stimulation and rest. During the stimulation periods, participants viewed a full-field flashing checkerboard at 3 Hz (∼80 ± 10 lux) and heard nonpainful tones ranging from 233.1 to 1318.5 Hz, presented at approximately 75 ± 5 dB, while performing a right-hand finger opposition task (touching the thumb sequentially to each fingertip). The first rest period lasted 20 seconds, followed by rest intervals of up to 30 seconds between blocks. After each stimulation block, participants rated the unpleasantness of the overall multisensory experience in the preceding block on a 0 to 100 computerized VAS, with 0 labeled as “not at all unpleasant” and 100 labeled as “the most unpleasant imaginable.”

All 126 participants also completed a resting-state fMRI scan during the same MRI session. Resting-state data were collected using the same orientation and slice geometry as the multisensory task (oblique, aligned to the orbitofrontal cortex; 57 slices; 2.5 mm^3^ voxel size), with a repetition time of 1500 milliseconds, echo time of 30 milliseconds at Stanford and 35 milliseconds at Cincinnati, flip angle = 70°, and field of view = 220 mm. Matrix sizes were 88 × 88 (Stanford) and 88 × 87 (Cincinnati), with a multiband factor of 3 and 257 volumes acquired per scan. In-plane acceleration was implemented using Acceleration phase = 2 on the GE Premier (Stanford) and SENSE = 1 on the Philips Ingenia Elition (Cincinnati). Participants were instructed to remain still with their eyes open and avoid falling asleep during the resting-state scan.

All fMRI data were processed using the CONN toolbox (version 22a; https://web.conn-toolbox.org) within MATLAB R2022a (https://matlab.mathworks.com). The first 6 volumes of each time series were discarded to allow magnetization to reach equilibrium. Realignment and unwarping were performed to correct for head motion.^32^ Outlier detection flagged volumes whose framewise displacement exceeded 0.9 mm or global BOLD signal change exceeded 5 standard deviations.^66^ Functional data were then spatially normalized to a standard Montreal Neurological Institute (MNI) template with 2 mm isotropic voxels.^5^ Finally, the normalized data were smoothed using a 6 mm full-width-at-half-maximum Gaussian kernel.^54^

For resting-state data, additional denoising was performed using aCompCor from the CONN toolbox, including white matter and CSF time series, 6 motion-parameter regressors, and regressors for outlier volumes. We applied a temporal band-pass filter of 0.008 to 0.09 Hz.

### 2.8. Statistics

Pain characteristics and self-report measure results were calculated and compared between the HD and CP groups using independent samples t-tests.

Quantitative sensory testing data were compared between the HD and CP groups. Missing values were treated as missing in the analysis and not imputed. Analyses were carried out using IBM SPSS Statistics (Version 29.0.2.0), JASP (Version 0.19.3), and R statistical software (R version 4.4.2 [2024-10-31]). Quantitative sensory testing results were first assessed for normality. As the data were nonnormally distributed, nonparametric Mann-Whitney U tests were conducted to compare group differences between those with and without a hypermobility disorder. To control for the study site (Stanford vs Cincinnati), we carried out a generalized linear model (GLM) with a gamma distribution and a log link to compare QST results between the HD and non-HD groups using the study site as a covariate. QQ plots were created to visualize the distribution fit (see supplemental digital content, Fig. S1, http://links.lww.com/PAIN/C495). A separate gamma GLM was conducted to measure the effect of hypermobility on the QST variables stratified by study site (see supplemental digital content, Fig. S2, http://links.lww.com/PAIN/C495). In this case, a Bonferroni correction was also carried out to adjust for multiple hypothesis testing. Furthermore, a sensitivity analysis was conducted with participants with a Beighton score of ≥ 6 (see supplemental digital content, Fig. S3, http://links.lww.com/PAIN/C495).

In first-level fMRI analyses, we computed GLMs for each participant to identify brain regions with significant task-evoked brain activation during the multisensory blocks, compared with the baseline condition. We included as nuisance regressors the mean framewise displacement (FD) as a single motion-related regressor, and 1 regressor for each outlier volume identified during preprocessing. Before conducting second-level analyses, we tested whether group status (hypermobility present vs hypermobility absent) was significantly associated with site (Stanford vs Cincinnati) using a chi-square test (*P* = 0.005).

To assess whether task-evoked brain activation differences were present at baseline (ie, before performing the task), we computed intrinsic functional connectivity measures during the resting state. Specifically, we conducted resting-state seed-to-region-of-interest (ROI) connectivity analyses using a cerebellar region (identified during task performance) as the seed. Connectivity was assessed with 6 ROIs from the CONN default network atlas, corresponding to the bilateral primary somatosensory regions, motor areas, and supplementary motor areas. Significance was determined using false discovery rate (FDR) correction, with connections considered significant at *q* < 0.05 and interpreted as trend-level at 0.05 < *q* < 0.10.

## 3. Results

### 3.1. Patient demographics and pain characteristics

As seen in Table 1, the HD (hypermobility present) and CP (hypermobility absent) groups were well matched on age, sex at birth, and pubertal development. Those with HD showed significantly higher pain duration and pain spread. They also reported significantly higher fear of pain, fatigue, and functional disability, as well as lower quality of life and sleep quality. However, they did not significantly differ in pain intensity, pain unpleasantness, or mood symptoms (ie, depression and anxiety) compared with the CP group.

**Table 1:** Demographics of study participants characterized by hypermobility status.

	Hypermobility present (n=72)	Hypermobility absent (n=71)	T	*P*
Age	15.8 (1.6)	15.9 (1.3)		
Pubertal development	3.6 (0.6)	3.6 (0.6)		
Sex assigned at birth				
Male	6.9% (5)	7.0% (5)		
Female	93.1% (67)	93.0% (66)		
Ethnicity				
Hispanic or Latino	6.9% (5)	22.5% (16)		
Not Hispanic or Latino	77.8% (56)	76.1% (54)		
Decline to state/no response	15.3% (11)	1.4% (1)		
Race				
American Indian/Alaskan Native	0% (0)	1.5% (1)		
Asian	1.4% (1)	8.8% (6)		
Black/African American	4.2% (3)	2.9% (2)		
White	68.1% (49)	69.1% (47)		
Multiracial	12.5% (9)	16.2% (11)		
Decline to state/no response	12.5% (9)	5.6% (4)		
Unknown	1.4% (1)	0% (0)		
Pain-related characteristics				
Pain duration (mo)	58.5 (43.0)	35.6 (33.3)	−3.6	<0.001**
Pain intensity	55.2 (20.2)	51.1 (20.0)	−1.2	0.24
Pain unpleasantness	59.7 (24.2)	53.7 (22.0)	−1.5	0.13
Pain spread	5.1 (2.2)	3.9 (2.1)	−3.4	<0.001**
Self-reported outcomes				
Pain interference	5.2 (2.0)	4.6 (2.2)	−1.6	0.11
Functional disability	26.7 (9.8)	23.0 (11.4)	−2.0	0.04*
Fear of pain	22.2 (7.2)	18.6 (8.2)	−2.8	0.007**
PROMIS fatigue	66.3 (10.4)	60.6 (13.2)	−2.9	0.005**
PROMIS depression	58.2 (10.1)	55.2 (13.4)	−1.5	0.14
PROMIS anxiety	52.4 (11.4)	50.1 (12.6)	−1.1	0.26
Quality of life	47.0 (14.8)	54.2 (16.7)	2.7	0.008**
Pain catastrophizing	20.7 (9.9)	24.2 (11.6)	−2.8	0.08
Sleep quality	3.3 (0.7)	3.6 (0.8)	2.7	0.008**

Demographics of study participants characterized by their hypermobility status. Age, pubertal development, pain-related characteristics, and self-reported outcomes are reported as averages with standard deviations in parentheses. Race and ethnicity are reported as a percentage of the sample with the number of participants in parentheses. Between-group comparisons of pain characteristics and self-reported outcomes are reported with T-statistics and *P*-values. Asterisks indicate statistical significance levels: *P < 0.05 (2-tailed), **P < 0.01 (2-tailed).

### 3.2. Quantitative sensory testing

**Figure 2.**
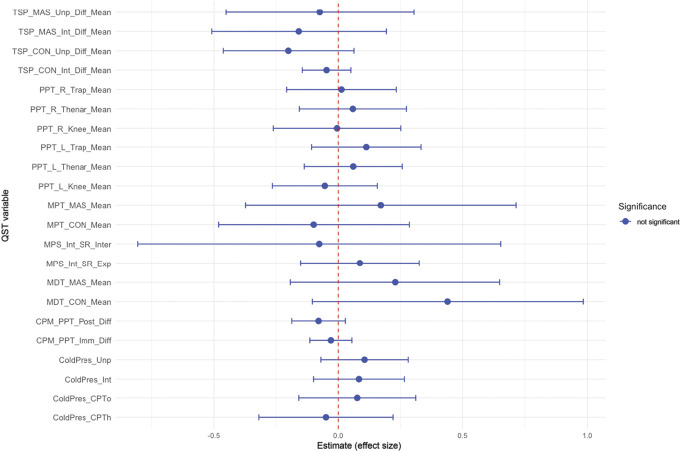
Gamma GLM of QST variables controlling for study site. MDT, mechanical detection threshold; MPT, mechanical pain threshold; MPS, mechanical pain sensitivity; PPT, pressure pain threshold; R, right; L, left; TSP, temporal summation of pain; CPM, conditioned pain modulation; control, dorsal hand; MAS, most affected site (patient's self-reported body region with the most pain); MPS_Int_SR_Inter, MPS intercept (VAS); MPS_Int_SR_Exp, MPS Slope (VAS); Unp, unpleasantness (VAS); Int, Intensity (VAS); CPM_PPT_Post_Diff, absolute CPM effect between baseline and post-immersion; CPM_PPT_Imm_Diff, absolute CPM effect between baseline and immersion. ColdPres_CPTh, cold pain threshold; ColdPres_CPTo, cold pain tolerance.

Quantitative sensory testing means stratified by group are reported in Table 2. In summary, no significant differences were found in either the Mann-Whitney U group comparisons (see Table S1, supplemental digital content, http://links.lww.com/PAIN/C495) or the gamma GLM controlling for the study site (Fig. 2). However, when gamma GLM was carried out to measure the effect of hypermobility on the QST variables stratified by study site, significant differences were found in the MDT at the Cincinnati study site but not at Stanford (see Fig. S2, supplemental digital content, http://links.lww.com/PAIN/C495). No other differences were found when stratified by the study site. The sensitivity analysis conducted to assess QST differences when removing participants with a Beighton score <6 (n = 6) did not affect the study results (see Fig. S3, supplemental digital content, http://links.lww.com/PAIN/C495).

**Table 2:** Quantitative sensory testing results.

QST test	Hypermobility present	Hypermobility absent
MDT control (mN)	8.7 (37.0)	2.3 (3.4)
MDT MAS (mN)	15.6 (39.9)	6.4 (6.8)
MPT control (mN)	126.0 (135.2)	134.5 (135.5)
MPT MAS (mN)	122.6 (158.4)	102.1 (146.9)
MPS slope (VAS)	2.0 (4.4)	1.9 (3.7)
MPS intercept (VAS)	4.1 (3.0)	3.7 (2.0)
PPT R trapezius (kPa)	172.0 (81.5)	176.6 (120.2)
PPT L trapezius (kPa)	174.0 (87.9)	162.1 (117.4)
PPT R thenar (kPa)	234.8 (105.2)	223.2 (144.2)
PPT L thenar (kPa)	241.7 (105.1)	222.9 (141.3)
PPT R knee (kPa)	265.2 (180.1)	257.4 (177.7)
PPT L knee (kPa)	241.7 (140.0)	245.6 (148.1)
TSP control intensity (VAS)	9.4 (11.8)	10.9 (12.2)
TSP control unpleasantness (VAS)	11.4 (13.1)	13.5 (13.3)
TSP MAS intensity (VAS)	13.8 (15.1)	14.5 (14.7)
TSP MAS unpleasantness (VAS)	15.6 (15.7)	15.8 (16.1)
CPM PPT immersion Δ (kPa)	−66.4 (53.4)	−59.3 (82.7)
CPM PPT post-immersion Δ (kPa)	−35.8 (58.3)	−24.2 (71.2)
Cold pressor threshold (s)	9.4 (7.2)	9.7 (7.7)
Cold pressor tolerance (s)	105.7 (68.8)	97.8 (68.0)
Cold pressor intensity (VAS)	55.2 (27.4)	49.8 (27.3)
Cold pressor unpleasantness (VAS)	64.0 (30.6)	56.0 (29.3)

QST results stratified by presence or absence of a hypermobility disorder. Values are reported as means with standard deviation in parentheses.

CPM PPT Δ refers to the change in PPT between baseline and immersion or post-immersion.

MDT, mechanical detection threshold (range: 0.63-235.36 mN); MPT, mechanical pain threshold (range: 8-512 mN); MPS, mechanical pain sensitivity (range: 8-512 mN); PPT, pressure pain threshold; R, right; L, left; TSP, temporal summation of pain; CPM, conditioned pain modulation; VAS, visual analog scale; control, Dorsal hand; MAS, most affected site (patient's self-reported body region with the most pain).

### 3.3. Task-related functional magnetic resonance imaging

**Figure 3.**
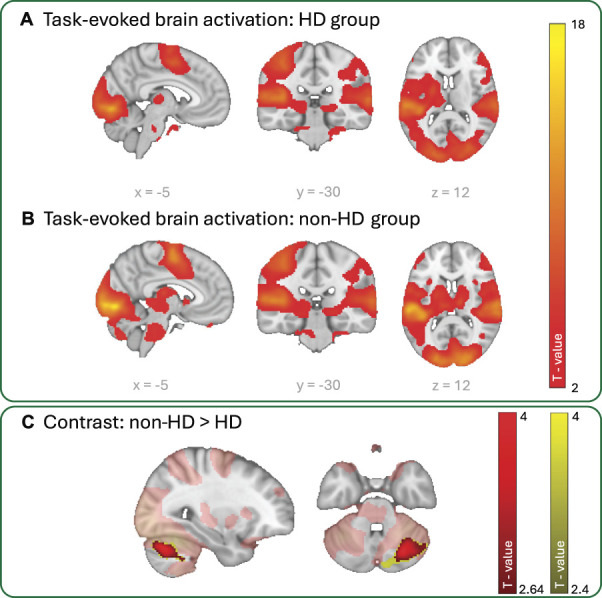
Comparison of task-evoked brain activation in individuals with and without hypermobility. (A) Task-evoked brain activation at the group level for individuals with hypermobility. (B) Task-evoked brain activation at the group level for individuals without hypermobility. (C) In red, the significant cluster identified for the contrast hypermobility absent > hypermobility present (*P* = 0.005 at voxel-level, cluster-level family-wise error-corrected *P* = 0.029). Regions in yellow are displayed at an uncorrected threshold of *P* < 0.01 for interpretative purposes. The color bars indicate T-values.

For the Stanford site, the contrast comparing hypermobility absent > hypermobility present revealed a significant cluster (cluster-level family-wise error correction with a corrected *P*-value of 0.029, *P*-voxel<0.005). This cluster encompassed 909 voxels and had a peak coordinate at (30, −68, −32) in MNI space, involving the right cerebellar hemisphere (ipsilateral to the finger opposition task), including the Right VI, Right Crus I, Right Crus II, and Right VIIIb according to the Diedrichsenlab probabilistic cerebellar atlas (Fig. 3).

No significant findings were observed in the Cincinnati site for either hypermobility present > hypermobility absent or hypermobility absent > hypermobility present contrasts.

We checked the temporal signal-to-noise ratio (tSNR) in the right cerebellar cluster (Fig. 3B) to assess and ensure signal quality. The mean tSNR was 150.4 for the multisensory task and 123.8 for the resting-state sequence. These values fall within the good-to-excellent range based on established benchmarks,^58,84^ confirming robust signal quality in this region (see supplemental digital content, Fig. S5, http://links.lww.com/PAIN/C495).

Furthermore, we tested whether group differences in cerebellar activation could reflect differences in motor output during the task. To assess this, we created 5-mm spherical ROIs centered on the finger representation of the bilateral primary motor cortices (MNI: ±32, −22, 68) and extracted average task-evoked activation (beta values) from these regions. We found no significant group differences in primary motor cortex activation in either hemisphere, in both the Stanford site (left: *P* = 0.215; right: *P* = 0.142) and the Cincinnati site (left: *P* = 0.658; right: *P* = 0.942). ROI masks and activation plots are provided in the Supplementary Materials (see Figs. S6–S7, supplemental digital content, http://links.lww.com/PAIN/C495).

**Figure 4.**
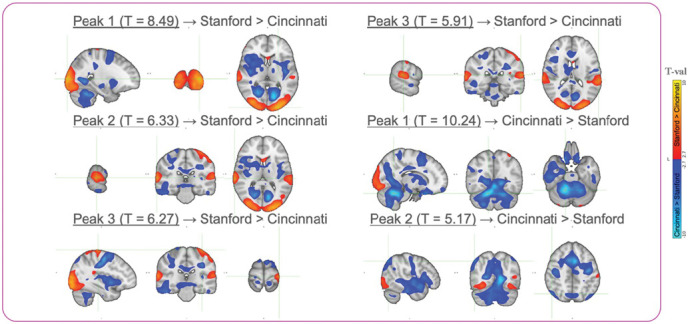
Between-site differences in brain activation. Site comparison results illustrating statistically significant clusters for Stanford > Cincinnati (red–orange) and Cincinnati > Stanford (blue), displayed on axial, coronal, and sagittal slices. Panels highlight principal peaks along with their respective T-values (in parentheses). Thresholding was applied at *P* < 0.005 at the voxel level, with clusters remaining significant under family-wise error correction at the cluster level. Color bars indicate T-values, ranging from positive (red–orange) to negative (blue).

In addition, to explore potential site-related effects, we compared task-evoked brain activation between participants scanned at Stanford and those scanned at Cincinnati. These analyses were conducted independently of the main comparisons (hypermobility present vs hypermobility absent). All results were thresholded at *P* < 0.005 at the voxel level, and significant clusters were assessed using family-wise error correction at the cluster level (Fig. 4).

We used the cerebellar region that showed reduced task-evoked activation in hypermobility-present patients as the seed to assess potential connectivity alterations with somatosensory-motor network regions during baseline (resting-state sequence preceding the task). In the Stanford site, this cerebellar region showed stronger resting-state connectivity with the left SMA (Cohen's *d* = 0.35, *p*FDR = 0.048) in hypermobility-present patients. In the Cincinnati site, we observed a similar trend with the right postcentral gyrus (Cohen's *d* = 0.31, *p*FDR = 0.095).

## 4. Discussion

The findings of this study provide a nuanced perspective on sensory and pain perception in adolescents with HD and CP, a population that remains understudied. Patients with HD tend to exhibit more central sensitization and poorer health in various domains. We aimed to discern whether these differences are intrinsic to hypermobility or reflect broader CP characteristics.

Although prior studies suggest that individuals with hypermobility experience lower pain thresholds and heightened sensitization compared with healthy controls,^9,68,73^ our results demonstrated no significant QST differences between CP and HD groups. One potential avenue for this exploration lies in the physiological and psychological components that underpin various chronic MSK pain disorders, which may independently affect pain processing. Systemic inflammation, muscle tension, and structural MSK irregularities might modulate pain responses, regardless of hypermobility.^93^ Indeed, our QST findings showed higher sensitization (eg, lower PPT) in *both* HD and CP groups compared with published QST values for healthy adolescents.^10,53,73^ Chronic pain and MSK pathology may interact to produce a distinct phenotype not fully captured when comparing hypermobility and non-hypermobility groups alone.^56^

In our sample, pediatric HDs were associated with increased pain spread, reduced quality of life, greater fear of pain, elevated fatigue, and lower sleep quality, as supported by previous literature.^57,59,63,72,91^ The combined effect of these may lead to a cycle where higher pain spread diminishes sleep quality and overall well-being.^31,38,70^ Lack of mood differences suggests hypermobility may not intensify psychosocial factors beyond other pain conditions.^29^ Therefore, targeted interventions for CP and HD may not need to prioritize anxiety/depression beyond what is already standard for those living with CP. However, it is crucial to address the heightened fear avoidance related to pain, which is likely compounded by their susceptibility to repetitive injuries and microtraumas,^12,16,43,45^ thereby influencing their overall pain experience and coping.

Behaviorally, we do not see differences in QST results despite self-reported functional deficits, prompting exploration of underlying mechanisms.

The neuroimaging findings may provide context for this. In the Stanford site, significant differences in cerebellar activation between hypermobile and non-hypermobile individuals during a multisensory fMRI task were revealed, particularly in regions such as Right VI, Crus I, Crus II, and VIIIb. These areas are well established as critical for sensorimotor integration, proprioception, cognitive functions (eg, working memory, executive processing), and pain-related activation.^6,20,90^ Interestingly, we found that hypermobile patients showed disrupted (ie, reduced) connectivity between these cerebellar regions and the supplementary motor cortex at baseline, preceding the onset of the fMRI multisensory task. These findings suggest that the functional organization of the cerebellar–cerebral motor circuit may be compromised in HD patients, potentially making them prone to hyperactivate the cerebellar region when task demands increase. To our knowledge, this is the first evidence linking hypermobility in the context of chronic pain to aberrant cerebellar–cerebral motor circuits. These results converge with evidence indicating the cerebellum plays a key role in coordinating sensory input and motor output, functions often reported as impaired in individuals with HD.^4,30,69,94^ This provides insight into the central nervous system's role in sensory-motor stimulus processing and response in HD, and highlights the importance of investigating cerebellar function as a potential contributor to the broader symptomatology in HD. It is feasible that proprioception and motor function deficits at the cerebellar level during such tasks could exacerbate proprioceptive challenges already inherent to HD^18^ and may contribute to the susceptibility of patients to recurrent microtraumas. This may lead to increased physical effort to maintain motor control, contributing to poorer quality of life, sleep quality, and fatigue. Deficits in working memory and demands on executive function may also perpetuate fatigue.^3,52^

To reconcile the QST and fMRI results, it is important to consider these modalities' distinct nature. QST measures pain thresholds and sensory stimulus perception in controlled, isolated conditions,^64^ while fMRI captures neural activation patterns during complex, integrative tasks that simulate real-world conditions.^79^ The similarity in QST results between the HD and CP groups may reflect the capacity of individuals with HD to reach similar levels of perception under controlled testing conditions despite potentially engaging in different neural strategies when faced with broader, more demanding task contexts. This discrepancy suggests that while both groups exhibit similar sensory detection capabilities, the underlying neural mechanisms and cognitive demands associated with processing sensory information can differ significantly. These findings emphasize the importance of a multifaceted approach when interpreting sensory processing in individuals with CP, as measures like QST may not fully capture the complexities of pain perception in real-life contexts. Furthermore, some critics note that QST is more of a measure of psychosocial functioning rather than underlying sensitization.^62^ Indeed, in our study, those with HD do not show differences in anxiety, depression, or pain catastrophizing compared with the CP cohort, which may help account for the lack of QST differences.

In summary, our results support that having a diagnosis of HD with CP does not increase sensitization compared with CP alone. However, we find that patients with HD experience greater deficits in daily functioning and overall well-being. We also see differences at the neural level during nonnoxious multisensory tasks that support that the impact of hypermobility on sensory processing extends beyond pain thresholds and may influence overall sensory integration, motor control, and the ability to adapt to sensory stimuli. These neural differences may also aid in explaining the functional deficits observed. Together, these findings are consistent with features of a nociplastic pain phenotype.

### 4.1. Limitations

There are limitations that warrant acknowledgment, including the heterogeneity inherent within groups classified as having hypermobility. Hypermobility can present in varying degrees of severity and can coexist with numerous psychosocial factors and other medical diagnoses.^17,22,35,44,81^ We attempted to limit this heterogeneity by restricting our hypermobile cohort to those with a Beighton score ≥4 and a diagnosis from the pediatric pain or hypermobility clinic. Notably, although HD diagnosis status did not significantly affect the QST results when accounting for the effect of the study site, the study site itself had a significant effect on MDT (ie, Cincinnati showed significantly higher MDT in the HD group). Therefore, important differences between the study sites may influence the measures. Although the QST examiners were rigorously trained, variability in testing is still likely to occur due to time of day, user error, ambient temperature (although the rooms are controlled), or individual differences in pain processing.^8,60^

The neuroimaging findings should be interpreted cautiously due to limitations related to site differences, statistical power, and scanner variability. Significant cerebellar activation differences were observed in the Stanford site, but these were not replicated in the Cincinnati site. Based on the effect size observed in the Stanford site (Hedges' *g* = 0.86), detecting this effect with 80% power using a conservative two-tailed test would require 22 participants per group. Although the Cincinnati site should have had sufficient statistical power for a directional replication attempt (1-tailed), the lack of replication combined with the smaller sample size suggests that these activation patterns may be influenced by site-specific scanner differences or cohort heterogeneity.

Site-related scanner variations in manufacturer, model, magnetic field strength, and acquisition protocols are known to influence image quality and derived measures, potentially masking biologically meaningful associations or introducing bias.^1^ Site-specific factors, such as environmental conditions during scanning and subtle differences in task administration, could also affect results. This is consistent with the strong between-site differences observed in the present study.

As another limitation, we did not collect device-based measures of motor output (eg, tap frequency or force), which restricts the ability to quantify task-related movement vigor. To indirectly address this, we conducted additional analyses, which showed no significant between-group differences in task-evoked activation of the primary motor cortices for the fingers.

Future studies should aim to increase sample sizes, standardize imaging protocols, and include objective behavioral measures to ensure reproducibility and interpretability of findings across sites. Although task-evoked fMRI identified neural differences in a well-powered cohort, the lack of replication in a smaller sample highlights the need for larger samples and complementary neurobiological approaches to fully characterize brain mechanisms associated with HD in adolescents with chronic pain.

### 4.2. Future directions

Several recommendations emerge for future research. First, longitudinal studies that track pain perception over time in individuals with hypermobility could illuminate how pain processing evolves and interacts with other physical, psychological, and developmental changes. Second, there is a need to implement the standardized diagnostic criteria described,^13,50,86^ as our chart review revealed that this diagnosis is complex. This is especially important in children and adolescents who are often nonpathologically hypermobile.^25^ Finally, research could benefit from mixed methods designs to incorporate quantitative alongside qualitative measures that explore individual narratives of pain. Understanding lived experiences could provide insights into how hypermobility influences pain beyond sensory processing metrics. These approaches may enhance our understanding of the interplay between HD and CP, paving the way for targeted treatments that address the physiological and psychosocial dimensions of this condition.

## Conflict of interest statement

The authors have no conflict of interest to declare.

## Supplemental digital content

Supplemental digital content associated with this article can be found online at http://links.lww.com/PAIN/C495.

## Supplementary Material
