# Supplementary material for: Hypermobility and chronic pain in adolescents: diverging functional and neural profiles without sensory differences
Source: Pain. 2026 May 15;167(8):e312–24. doi: 10.1097/j.pain.0000000000003999 (PMC13382870; doi:10.1097/j.pain.0000000000003999)
Supplement: Supplementary file 1 [file jop-167-e312-s001.pdf]

Table S1. Demographics Stratified by Study Site

	Stanford Cohort		Cincinnati Cohort	
	Hypermobility Present (n=36)	Hypermobility Absent (n=52)	Hypermobility Present (n=36)	Hypermobility Absent (n=19)
Age	15.9 (1.6)	16.0 (1.2)	15.7 (1.6)	15.6 (1.5)
Pubertal Development	3.6 (0.3)	3.7 (0.6)	3.5 (0.7)	3.5 (0.6)
Sex Assigned at Birth				
Male	8.3% (3)	7.7% (4)	5.6% (2)	5.3% (1)
Female	91.7% (33)	92.3% (48)	94.4% (34)	94.7% (18)
Ethnicity				
Hispanic or Latino	11.1% (4)	30.8% (16)	2.8% (1)	0% (0)
Not Hispanic or Latino	77.8% (28)	69.2% (36)	77.8% (28)	94.7% (18)
Decline to State/No Response	11.1% (4)	0% (0)	19.4% (7)	5.3% (1)
Race				
American Indian/Alaskan Native	0% (0)	1.9% (1)	0% (0)	0% (0)
Asian	2.8% (1)	11.5% (6)	0% (0)	0% (0)
Black/African American	2.8% (1)	1.9% (1)	5.6% (2)	5.3% (1)
White	72.2% (26)	59.6% (31)	63.9% (23)	84.2% (16)
Multiracial	13.9% (5)	19.2% (10)	11.1% (4)	5.3% (1)
Decline to State/No Response	5.6% (2)	5.8% (3)	19.4% (7)	5.3% (1)
Unknown	2.8% (1)	0% (0)	0% (0)	0% (0)

Demographics of study participants characterized by their hypermobility status and stratified by study site. Age, pubertal development, pain related characteristics, and self-reported outcomes are reported as averages with standard deviations in parentheses. Race and ethnicity are reported as percentage of sample with number of participants in parentheses.

Table S2. Normality-Violating Pain Characteristics Between-Group Comparisons

	p	U
Pain Duration	1,634	< .001
Pain Spread	1,734	< .001
Pain Interference	2,155	.136
PROMIS Fatigue	1,861	.014
PROMIS Depression	2,104	.150
PROMIS Anxiety	2,216	.329
Pain Catastrophizing	2,099	.112
Pain Unpleasantness	2,023	.099

Between-group comparisons of pain characteristics between participants with HD and participants without HD using Mann-Whitney U non-parametric tests. These pain characteristics violated the assumption of normality based Shapiro-Wilk tests.

Table S3. Quantitative Sensory Testing Group Comparisons

	Hypermobility Present	Hypermobility Absent	U	p
TSP MAS Unpleasantness (VAS)	15.6 (15.7)	15.8 (16.1)	1310.000	0.952
TSP MAS Intensity (VAS)	13.9 (15.1)	14.5 (14.7)	1347.500	0.756
TSP Control Unpleasantness (VAS)	11.4 (13.1)	13.5 (13.3)	2810.500	0.236
TSP Control Intensity (VAS)	9.3 (11.8)	10.9 (12.2)	2657.000	0.577
PPT R Trap (kPa)	172.0 (81.5)	176.6 (120.2)	1368.000	0.312
PPT R Thenar (kPa)	234.8 (105.2)	223.2 (144.2)	1318.500	0.247
PPT R Knee (kPa)	265.2 (105.1)	257.4 (177.7)	1488.500	0.764
PPT L Trap (kPa)	174.0 (87.9)	162.1 (117.4)	1505.500	0.069
PPT L Thenar (kPa)	241.7 (105.1)	222.9 (141.3)	1552.000	0.115
PPT L Knee (kPa)	241.7 (140.0)	245.6 (148.1)	1837.000	0.906
MPT MAS (mN)	122.6 (158.4)	102.1 (146.9)	1136.000	0.211
MPT control (mN)	126.0 (135.2)	134.5 (135.5)	1794.500	0.651
MPS Slope (VAS)	2.0 (4.4)	1.9 (3.4)	1794.000	0.653
MPS Intercept (VAS)	4.1 (3.0)	3.7 (2.0)	1716.000	0.980
MDT MAS (mN)	10.2 (11.1)	6.4 (6.8)	1082.500	0.082
MDT control (mN)	3.9 (5.5)	2.3 (3.4)	1540.000	0.436
CPM PPT Post-Immersion Δ (kPa)	-36.8 (58.3)	-24.2 (71.2)	2570.000	0.420
CPM PPT Immersion Δ (kPa)	-66.4 (53.4)	-59.3 (82.7)	2664.000	0.293
Cold Pressor Unpleasantness (VAS)	64.0 (30.6)	56.0 (29.3)	1921.000	0.089
Cold Pressor Intensity (VAS)	55.2 (27.4)	49.8 (27.3)	2058.000	0.271
Cold Pressor Tolerance (sec)	105.7 (68.8)	97.8 (68.0)	2148.000	0.464
Cold Pressor Threshold (sec)	9.4 (7.2)	9.7 (7.7)	2315.500	0.988

Between-group comparison of QST test averages between HD and other chronic pain participants. QST results are reported as means with standard deviation in parentheses. MDT = Mechanical Detection Threshold (range: 0.63-235.36 mN); MPT = Mechanical Pain Threshold (range: 8-512 mN); MPS = Mechanical Pain Sensitivity (range: 8-512 mN); PPT = Pressure Pain Threshold; R = Right; L = Left; TSP = Temporal Summation of Pain; CPM = Conditioned Pain Modulation; VAS = Visual Analog Scale; Control = Dorsal hand; MAS = Most Affected Site (patient's self-reported body region with the most pain). Mann-Whitney U statistics and p-values are reported.

Table S4. HD diagnoses

Diagnosis	ICD-10 Code	Frequency
Hypermobile joint syndrome;<br>Generalized hypermobility of joints	M24.80	5.6% (4)
Hypermobile joints;<br>Hypermobility of joint	M24.9	2.8% (2)
Hypermobility arthralgia	M25.50	13.9% (10)
Hypermobility syndrome;<br>Benign joint hypermobility;<br>Benign hypermobility syndrome;<br>Benign joint hypermobility syndrome	M35.7	40.3% (29)
Ehlers-Danlos syndrome;<br>Ehlers-Danlos disease	Q79.60	11.1% (8)
Ehlers-Danlos, hypermobile type;<br>Hypermobile Ehlers-Danlos syndrome;<br>Ehlers-Danlos, benign hypermobile form;<br>Ehlers-Danlos syndrome	Q79.62	45.8% (33)
Beighton Score	Range	Mean
	4-9	6.7

Diagnoses, ICD-10 codes, and Beighton scores of participants in the HD group. Diagnostic frequencies are reported as percentage of sample with number of participants in parentheses. Participant frequencies add up to more than 100%, as some participants held multiple diagnoses. The range and mean of Beighton scores are reported.

Table S5. Diagnostic categories characterized by HD status

Diagnostic Category	**Hypermobility Present**	**Hypermobility Absent**
Localized Musculoskeletal Pain	23.6% (17)	43.1% (31)
Abdominal Pain	16.7% (12)	9.7% (7)
Headache	11.1% (8)	19.4% (14)
Generalized Pain Syndromes	55.6% (40)	62.0% (44)
Neuropathic Pain	9.7% (7)	9.7% (7)

Representation of diagnoses present in the HD and non-HD cohorts. Frequencies are reported as percentage of sample with number of participants in parentheses.

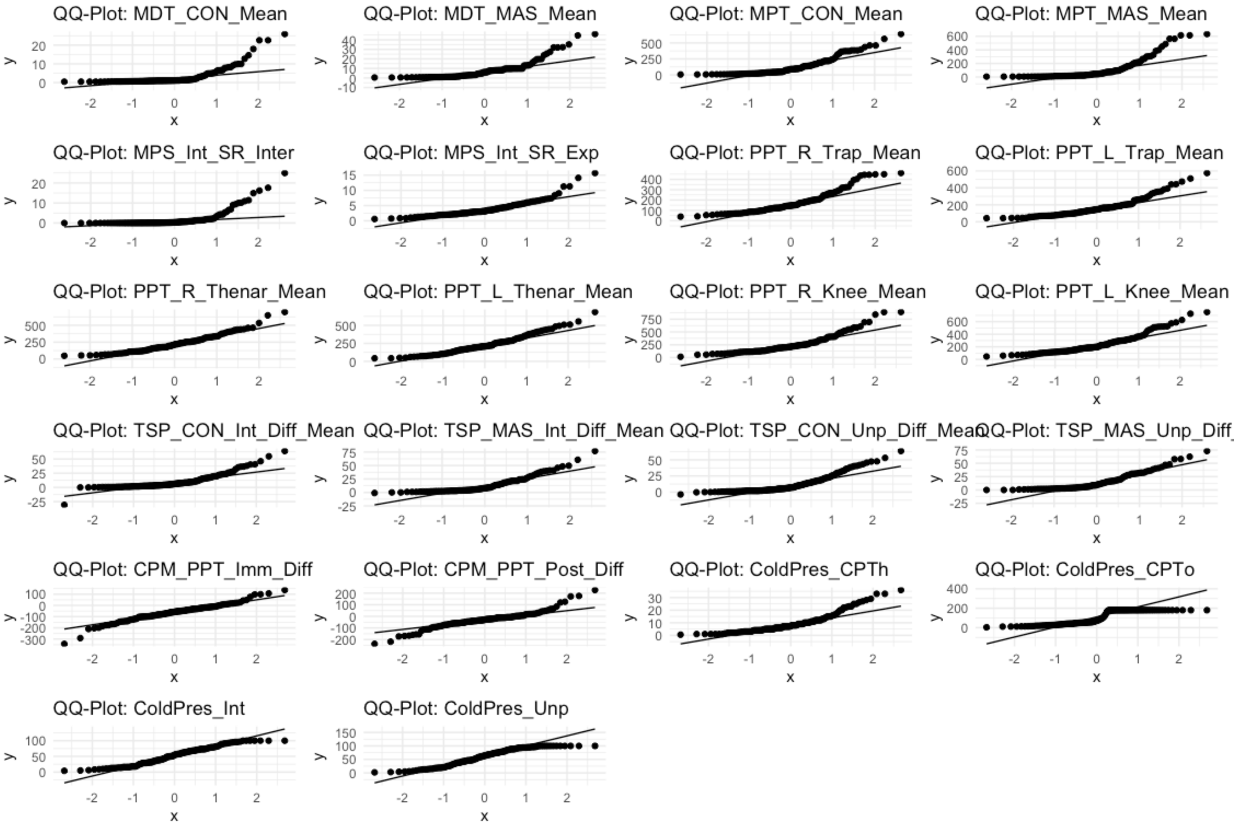

Figure S1. QQ Plots visualizing distribution fit of QST measures.
MDT = Mechanical Detection Threshold; MPT = Mechanical Pain Threshold; MPS = Mechanical Pain Sensitivity; PPT = Pressure Pain Threshold; R = Right; L = Left; TSP = Temporal Summation of Pain; CPM = Conditioned Pain Modulation; Control = Dorsal hand; MAS = Most Affected Site (patient's self-reported body region with the most pain); MPS_Int_SR_Inter = MPS intercept (VAS); MPS_Int_SR_Exp = MPS Slope (VAS); Unp = Unpleasantness (VAS); Int = Intensity (VAS); CPM_PPT_Post_Diff = Absolute CPM effect between baseline and post-immersion; CPM_PPT_Imm_Diff = Absolute CPM effect between baseline and during immersion. ColdPres_CPTh = Cold Pain Threshold; ColdPres_CPTo = Cold Pain Tolerance

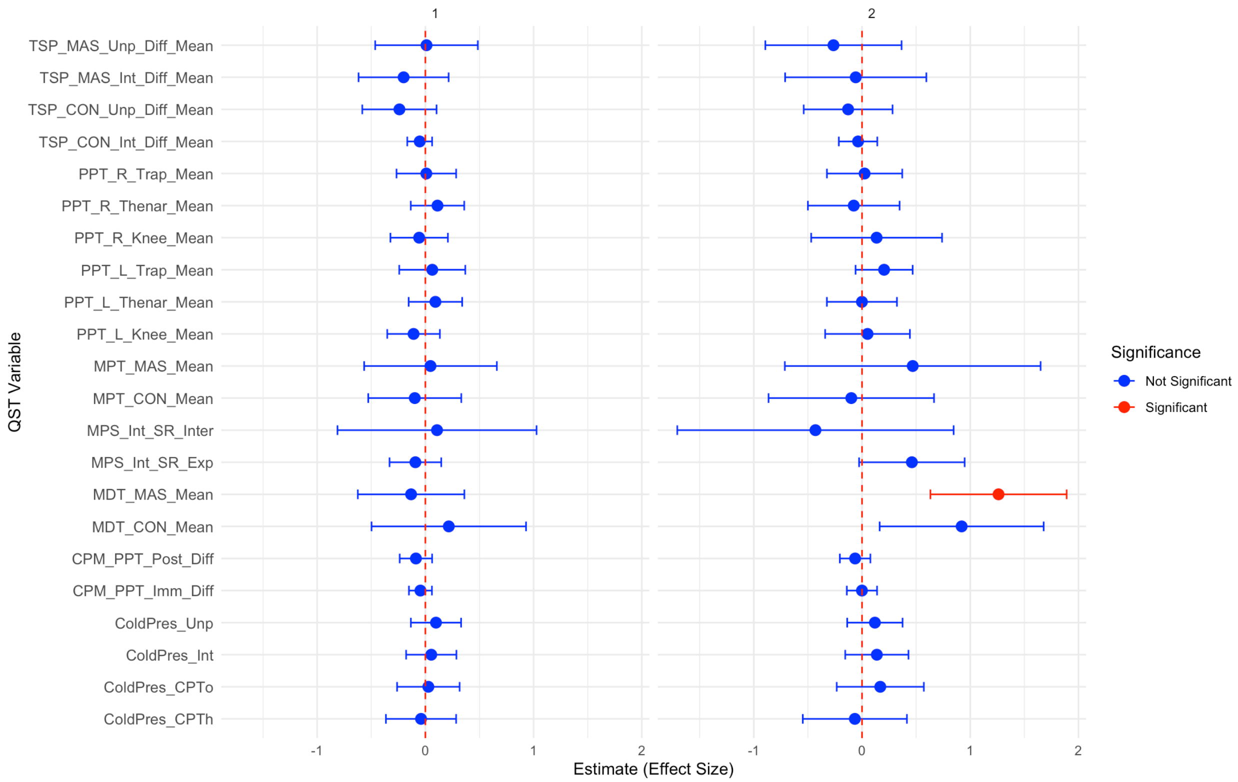

Figure S2. Gamma GLM with Bonferroni correction illustrating the effect of HD diagnosis on QST results stratified by study site (1= Stanford, 2= Cincinnati).

MDT = Mechanical Detection Threshold; MPT = Mechanical Pain Threshold; MPS = Mechanical Pain Sensitivity; PPT = Pressure Pain Threshold; R = Right; L = Left; TSP = Temporal Summation of Pain; CPM = Conditioned Pain Modulation; Control = Dorsal hand; MAS = Most Affected Site (patient's self-reported body region with the most pain); MPS_Int_SR_Inter = MPS intercept (VAS); MPS_Int_SR_Exp = MPS Slope (VAS); Unp = Unpleasantness (VAS); Int = Intensity (VAS); CPM_PPT_Post_Diff = Absolute CPM effect between baseline and post-immersion; CPM_PPT_Imm_Diff = Absolute CPM effect between baseline and during immersion. ColdPres_CPTh = Cold Pain Threshold; ColdPres_CPTo = Cold Pain Tolerance

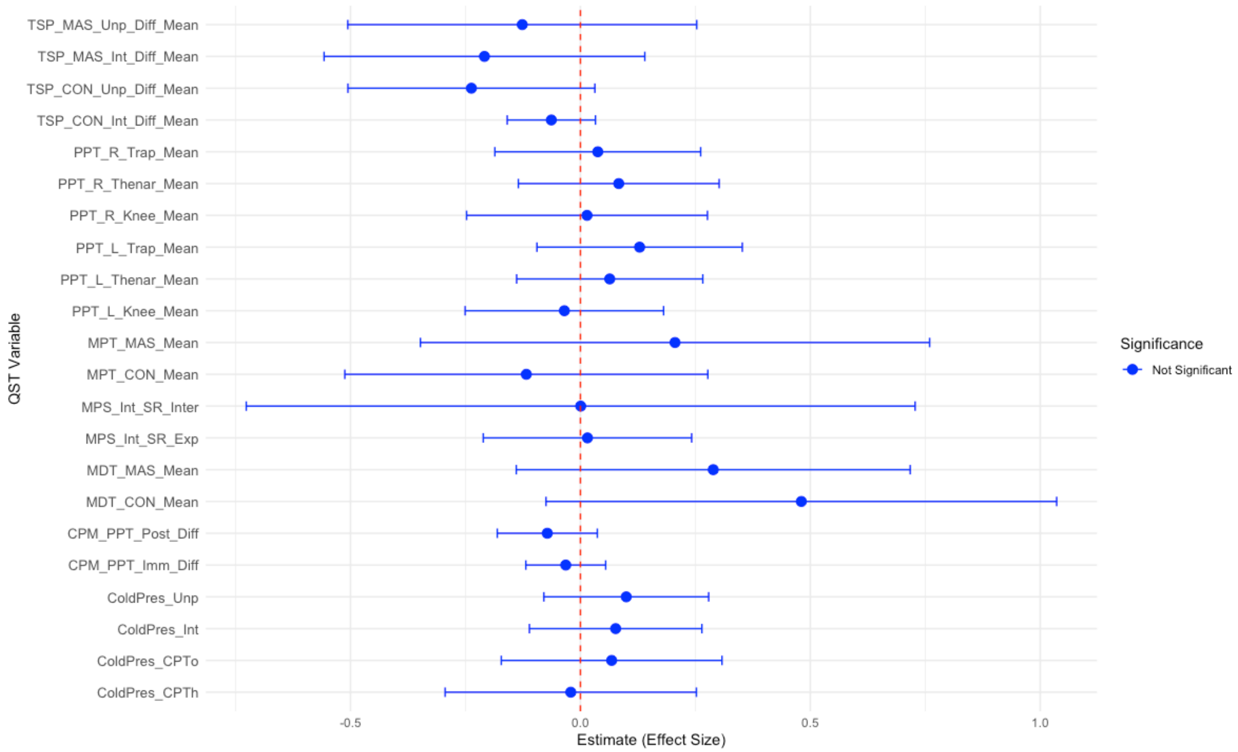

Figure S3. Gamma GLM with Bonferroni correction of QST measures illustrating the sensitivity analysis performed when participants with Beighton score of <6 are removed from analysis.
MDT = Mechanical Detection Threshold; MPT = Mechanical Pain Threshold; MPS = Mechanical Pain Sensitivity; PPT = Pressure Pain Threshold; R = Right; L = Left; TSP = Temporal Summation of Pain; CPM = Conditioned Pain Modulation; Control = Dorsal hand; MAS = Most Affected Site (patient's self-reported body region with the most pain); MPS_Int_SR_Inter = MPS intercept (VAS); MPS_Int_SR_Exp = MPS Slope (VAS); Unp = Unpleasantness (VAS); Int = Intensity (VAS); CPM_PPT_Post_Diff = Absolute CPM effect between baseline and post-immersion; CPM_PPT_Imm_Diff = Absolute CPM effect between baseline and during immersion. ColdPres_CPTh = Cold Pain Threshold; ColdPres_CPTo = Cold Pain Tolerance

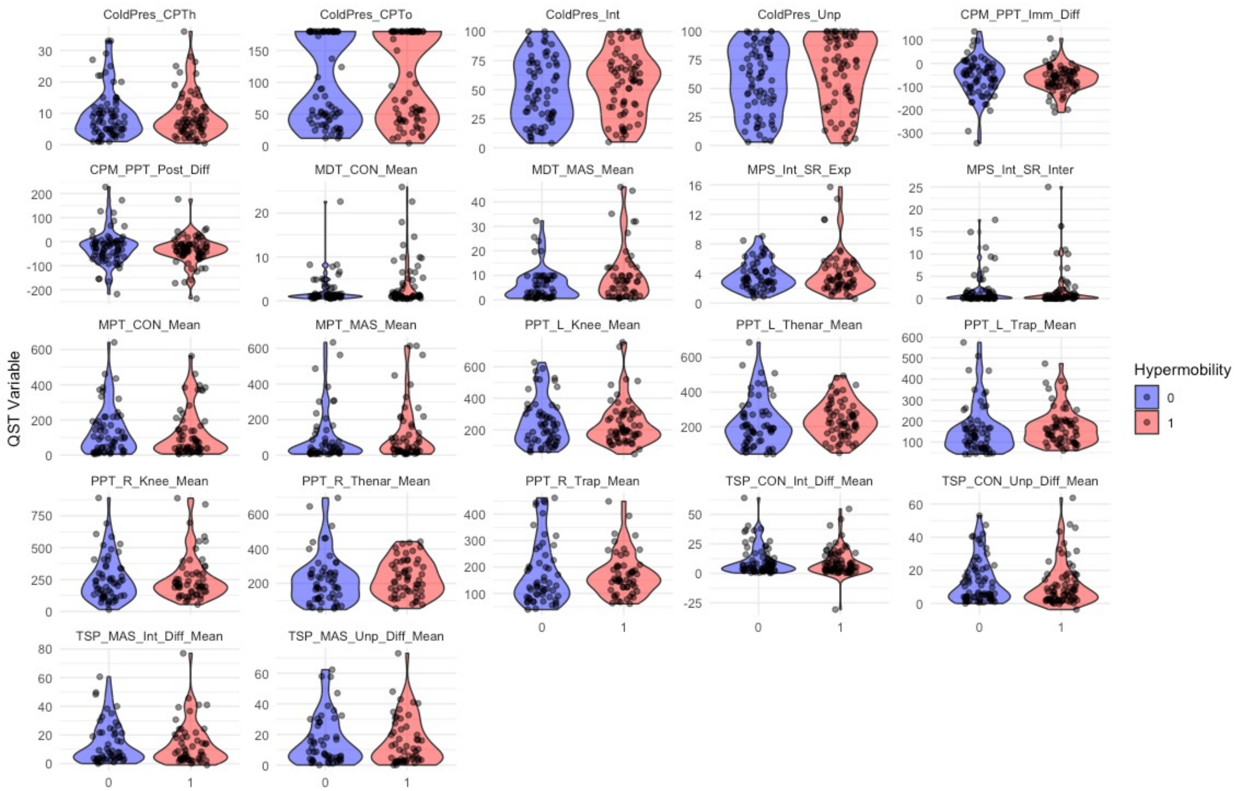

Figure S4. Violin plots exhibiting distribution of QST variables stratified by hypermobility.
MDT = Mechanical Detection Threshold; MPT = Mechanical Pain Threshold; MPS = Mechanical Pain Sensitivity; PPT = Pressure Pain Threshold; R = Right; L = Left; TSP = Temporal Summation of Pain; CPM = Conditioned Pain Modulation; Control = Dorsal hand; MAS = Most Affected Site (patient's self-reported body region with the most pain); MPS_Int_SR_Inter = MPS intercept (VAS); MPS_Int_SR_Exp = MPS Slope (VAS); Unp = Unpleasantness (VAS); Int = Intensity (VAS); CPM_PPT_Post_Diff = Absolute CPM effect between baseline and post-immersion; CPM_PPT_Imm_Diff = Absolute CPM effect between baseline and during immersion. ColdPres_CPTh = Cold Pain Threshold; ColdPres_CPTo = Cold Pain Tolerance. Hypermobility 0 (purple) = non-HD group; Hypermobility 1 (red) = HD group.

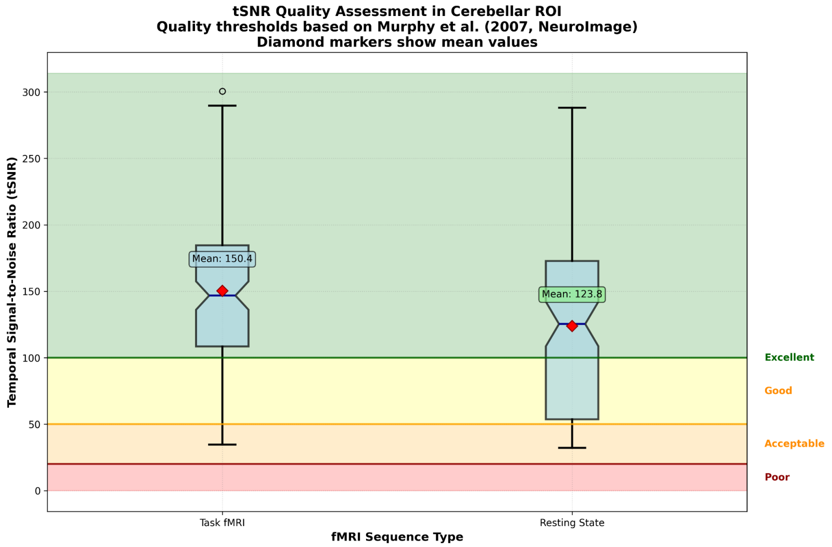

Figure S5. Temporal signal-to-noise ratio (tSNR) in the cerebellar cluster

Violin plots displaying the distribution of tSNR values for both the multisensory task and resting-state sequences within the right cerebellar cluster (MNI: 30, –68, –32). Diamond markers indicate the mean tSNR for each sequence (task = 150.4; rest = 123.8). Background shading denotes standard tSNR quality bands, indicating that signal quality was within the good-to-excellent range (Murphy et al., 2007; Taylor et al., 2024).

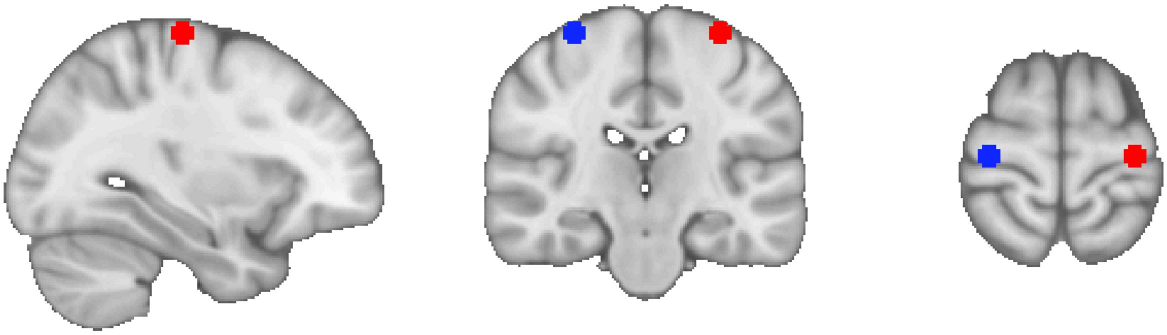

Figure S6. Finger-specific M1 ROI masks
Illustration of bilateral 5-mm spherical ROIs centered on the finger representation of the primary motor cortex (MNI: ±32, –22, 68). ROIs were used to extract task-evoked activation for the purpose of assessing potential group differences in motor output during the multisensory task.

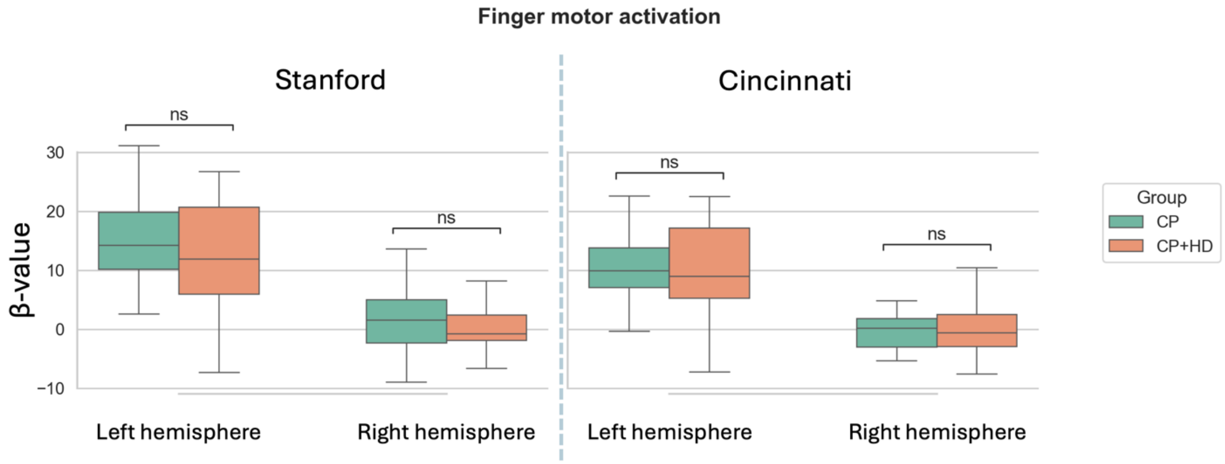

Figure S7. Task activation in left and right M1 finger ROIs by group and site

Boxplots showing task-evoked activation (β values) in the finger-specific M1 ROIs for each group (CP and CP + HD) and each cohort (Stanford and Cincinnati). No significant group differences were found in either hemisphere or cohort (all $p > 0.1$).
